# Outcomes of Gamma Knife Radiosurgery for Brain Metastases From Anaplastic Lymphoma Kinase Rearrangement-Positive and EGFR Mutation-Positive Non-Small Cell Lung Cancer

**DOI:** 10.7759/cureus.20398

**Published:** 2021-12-13

**Authors:** Shigeo Matsunaga, Takashi Shuto

**Affiliations:** 1 Department of Neurosurgery, Yokohama Rosai Hospital, Yokohama, JPN

**Keywords:** stereotactic radiosurgery, lung cancer, gamma knife, epidermal growth factor receptor, driver gene mutation, brain metastases, anaplastic lymphoma kinase

## Abstract

Introduction

The outcomes after gamma knife radiosurgery (GKRS) were retrospectively analysed in patients with brain metastases from anaplastic lymphoma kinase (ALK) rearrangement-positive and epidermal growth factor receptor (EGFR) mutation-positive non-small cell lung cancer (NSCLC) to evaluate the efficacy, safety and difference for overall survival and local tumor control.

Methods

The medical records were retrospectively reviewed of 607 patients (25 ALK-positive, 171 EGFR-positive, and 411 wild type) with 2959 tumors who had undergone GKRS.

Results

The median overall survival time after initial GKRS was 14 months. Driver gene mutation-positive patients had significantly longer overall survival than wild type patients (p < 0.0001), and ALK-positive patients survived significantly longer than EGFR-positive patients (p = 0.04). Multivariate analysis showed the unfavorable factors significantly affecting overall survival outcomes were older age, lower Karnofsky Performance Status score, multiple intracranial metastases, uncontrolled primary cancer, uncontrolled extracranial metastases, no administration of immune checkpoint inhibitors, and driver gene mutation-negative cases. Seventy-three patients died of uncontrolled brain metastases at a median of 12 months. Driver gene mutations had no influence (p = 0.33), and ALK-positive and EGFR-positive patients showed no significant difference in neurological survival (p = 0.83). A total of 174 patients demonstrated distant brain control failure at a median of 15 months. ALK-positive type was significant compared with EGFR-positive type (p = 0.047), but driver gene mutation-positive and -negative types showed no significant difference in the development of new brain metastases (p = 0.2). The median tumor volume was 1.06 cm^3^ in the driver gene mutation-positive type and 1.85 cm^3^ in wild type. The median marginal dose was 20 Gy in both types. The 6-, 12-, and 24-month local tumor control rates were 97.3%, 96.1%, and 95.9%, respectively. Driver gene mutations had a significantly positive impact on local tumor control (p = 0.001), and ALK-positive and EGFR-positive types showed no significant difference (p = 0.95). A total of 193 tumors had radiation injury at a median of 12 months after GKRS. The 6-, 12-, and 24-month GKRS-related complication rates were 3.3%, 8.1%, and 8.7%, respectively. Driver gene mutations significantly induced radiation damage (p = 0.021), and the ALK-positive type was affected more than the EGFR-positive type (p = 0.02).

Conclusions

ALK rearrangement-positive NSCLC patients tended to have significantly longer survival, but had higher incidence of new intracranial metastases due to long-term survival after GKRS, compared with EGFR mutation-negative and driver gene mutation-negative NSCLC patients. GKRS induced significantly satisfactory local tumor control in driver gene mutation-positive tumors but GKRS-related complication frequency was higher, especially in ALK-positive NSCLC patients. Therefore, more careful imaging follow-up is necessary after GKRS for patients with driver gene mutation-positive NSCLC.

## Introduction

Epidermal growth factor receptor (EGFR) mutations are more common in Japanese than in Western patients with non-small cell lung cancer (NSCLC). EGFR mutations are positive in 50% of Japanese patients with NSCLC, while anaplastic lymphoma kinase (ALK) rearrangement is positive in only 3%-5% [1,2]. Many new drugs have been developed for the treatment of patients with positive driver gene mutations, of which the third generation of tyrosine kinase inhibitors (TKIs) for ALK and EGFR are currently approved, and more new drugs are expected to be introduced in the future [3].

NSCLC is found at the highest proportion of primary sites of metastatic brain tumors. Brain metastases from NSCLC are treated, with single or multimodality therapies including surgical resection, whole-brain radiation therapy (WBRT), and stereotactic radiosurgery or radiotherapy, depending on the systemic condition of the patient. TKIs are especially effective against brain metastases in patients with positive driver gene mutation, such as ALK rearrangement or EGFR mutation [4,5]. However, various problems such as the acquisition of drug resistance are known [1,3,6-8]. Consequently, the desirable treatment to produce the optimum results has not been established. Radiosurgery is less invasive and effective against metastatic brain tumors from various primary cancers based on the palliation of neurological symptoms [9]. Radiosurgery has been used to treat brain metastases from EGFR mutation-positive NSCLC [10-12], and only a small number of cases of brain metastases from ALK rearrangement-positive cases; its efficacy and safety are less clear despite the reliable number of patients [10].

The present study retrospectively analysed the therapeutic efficacy and safety of gamma knife radiosurgery (GKRS) for the treatment of brain metastases from NSCLC, especially ALK rearrangement-positive tumors, compared with EGFR mutation-positive and driver mutation-negative wild type tumors.

## Materials and methods

Patient population

This population included a total of 607 patients with metastatic brain tumors from NSCLC, who had undergone single-dose GKRS at Yokohama Rosai Hospital after March 2012, when crizotinib, a first-generation ALK-TKI, was approved in Japan for the treatment of unresectable ALK rearrangement-positive advanced or recurrent NSCLC. Major criteria for inclusion were as follows: (1) driver gene testing had been performed for the NSCLC; (2) newly diagnosed brain metastasis, and GKRS was the first treatment for brain metastasis; (3) treatment or follow-up of the primary tumor, and extracranial metastases was ongoing at the time of GKRS; (4) Karnofsky Performance Status (KPS) score of 70% or higher before the onset of neurological symptoms due to brain metastasis; (5) tumor volume of the largest lesion was 10 cm^3^ or less; and (6) T1-weighted gadolinium-enhanced magnetic resonance (MR) imaging at the time of initial treatment found no obvious findings of meningeal carcinomatosis. We obtained approval from our local institutional review board for this study (Yokohama Rosai Hospital IRB approval 31-17). Treatment outcome data were collected by the first author (SM).

GKRS techniques

Stereotactic radiosurgery was performed with the Leksell Gamma Knife® Perfexion™ system (Elekta Instrument AB, Stockholm, Sweden). The Leksell G-frame was placed under local anesthesia. Intravenous anesthesia was also used to reduce pain and anxiety during pin fixation. The Leksell Gamma Plan system (Elekta Instrument AB) was used for dose planning based on computed tomography scans and T1-weighted gadolinium-enhanced MR images with 1-mm slice thickness with no gaps. Only single-dose irradiation was included, and fractionated irradiation was excluded. Prescribed doses were based on the target volume. Post-GKRS clinical and radiographic examinations were obtained from the patients and their referring physicians.

Post-GKRS follow-up protocol

All patients underwent periodic clinical and neuroradiological follow-up examinations after the initial GKRS. The radiographic response was evaluated based on the changes in the maximum diameter of the irradiated tumors on post-GKRS MR images in accordance with the revised Response Evaluation Criteria in Solid Tumours (RECIST) guidelines [13]. The maximum diameter of the target tumor was measured on axial, coronal, and sagittal MR images for the assessment of the tumor size. The GKRS effects were classified into four categories: complete response (complete disappearance of the tumor), partial response (more than 50% decrease in tumor size), stable disease (less than 50% decrease and less than 25% increase in tumor size), and progressive disease (more than 25% increase in tumor size). Complete response, partial response, and stable disease were regarded as suppression of tumor growth. Tumor regrowth was differentiated from radiation injury using T1- and T2-weighted MR imaging in all cases. However, if this differential diagnosis was difficult based on only MR images, semi-quantitative analysis of thallium-201 single-photon emission computed tomography was used [14]. Symptomatic treatment-related toxicities were recorded, documented using the Common Terminology Criteria for Adverse Events (CTCAE) version 5.0, produced by the U.S. National Cancer Institute, Cancer Therapy Evaluation Program (http://ctep.cancer.gov), and CTCAE grade 2 or worse was judged as adverse effects of GKRS.

Clinical outcomes

The post-GKRS endpoints were overall survival, neurological death, distant brain control failure, local recurrence of the treated tumors, and irradiation-induced complications. For each endpoint, failures were regarded as events, and any others were censored. The overall survival time was defined as the interval between GKRS and death due to any cause or the day of the last follow-up examination. To estimate post-GKRS overall survival for each patient, the Diagnosis-Specific Graded Prognostic Assessment (DS-GPA) with the lung cancer molecular data (Lung-molGPA), and the modified Radiation Therapy Oncology Group recursive partitioning analysis (M-RPA) system were used [15,16]. Neurological death was defined as patient death related to uncontrolled progression of brain metastases, such as tumor recurrence, carcinomatous meningitis, or cerebral dissemination with leptomeningeal spread at the last follow-up examination. Distant brain control was defined as the absence of new distant lesions in the brain outside the previously irradiated area on neuroimaging after GKRS.

Statistical analysis

Summary statistics for baseline variables were constructed using frequencies and proportions for categorical data with median, inter-quartile range (IQR), and ranges for continuous variables. The standard Kaplan-Meier method was used for overall survival and local tumor control, and their cumulative incidences were compared using the log-rank test and the Cox proportional hazards model. In addition, to identify baseline and clinical variables associated with the outcome categories, except for overall survival and local tumor control, competing risk analyses were performed with Fine-Gray generalization of the proportional hazards model, accounting for death as a competing risk. The adjusted proportional hazards model was applied to identify the factors influencing survival and cumulative control rates with hazard ratio (HR) and 95% confidence interval (CI). Fisher’s exact test was used to identify the clinical characteristic and radiosurgical parameter differences between the driver gene mutation-positive and wild types. Statistical significance was set at p < 0.05 for a bilateral test. All statistical analyses were performed with EZR (Kanda Y, Saitama Medical Center, Jichi Medical University, 2012; freely available at http://www.jichi.ac.jp/saitama-sct/SaitamaHP.files/statmedEN.html), which is a graphical user interface for R version 2.13.0 (The R Foundation for Statistical Computing, Vienna, Austria). More precisely, this software is a modified version of R commander designed to add statistical functions frequently used in biostatistics.

## Results

Clinical outcomes of GKRS

Patient characteristics at initial GKRS are presented in Table 1 for the entire population.

**Table 1:** Clinical characteristics of patients with GKRS-treated brain metastases from non-small cell lung cancer ALK, anaplastic lymphoma kinase; EGFR, epidermal growth factor receptor; GKRS, gamma knife radiosurgery; IQR, interquartile range; KPS, Karnofsky Performance Status; M-RPA, modified Radiation Therapy Oncology Group recursive partitioning analysis; IQR, inter-quartile range *Fisher’s exact test.

Characteristics	Value		
	Driver gene mutation-positive type	Wild type	p-value*
No. of patients	196 (ALK 25/EGFR 171)	411	
Sex, male/female, n	79/117	275/136	<0.0001
Age			
Median (range, IQR), years	70 (40-92, 65-77)	70 (32-84, 65-76)	
≥70/<70 years, n	144/52	301/110	0.663
KPS score			
Median (range, IQR), %	100 (50-100, 90-100)	100 (40-100, 90-100)	
<70%/≥70%, n	19/177	47/364	0.579
No. of tumors at GKRS			
Median (range, IQR)	3 (1-14, 1-7)	2 (1-10, 1-5)	
≥5/<5 tumors, n	81/115	132/279	0.029
Neurological status, symptomatic/asymptomatic, n	28/168	96/315	0.009
Primary cancer controlled, no/yes, n	49/147	224/187	<0.0001
Extracranial metastases, active/controlled, n	123/73	272/139	0.414
Immune checkpoint inhibitor, no/yes, n	183/13	368/43	0.136
Interval time between primary diagnosis and GKRS			
Median (range, IQR), months	18 (0-62, 10-35)	7 (0-42, 0-18)	
Synchronous/metachronous, n	30/166	171/240	<0.0001
Lung-molGPA, n			<0.0001
3.5-4.0	56	0	
2.5-3.0	92	101	
1.5-2.0	44	219	
0.0-1.0	4	91	
M-RPA, n			0.001
Class 1 + 2a	99	143	
Class 2b	41	87	
Class 2c + 3	56	181	

**Figure 1:**
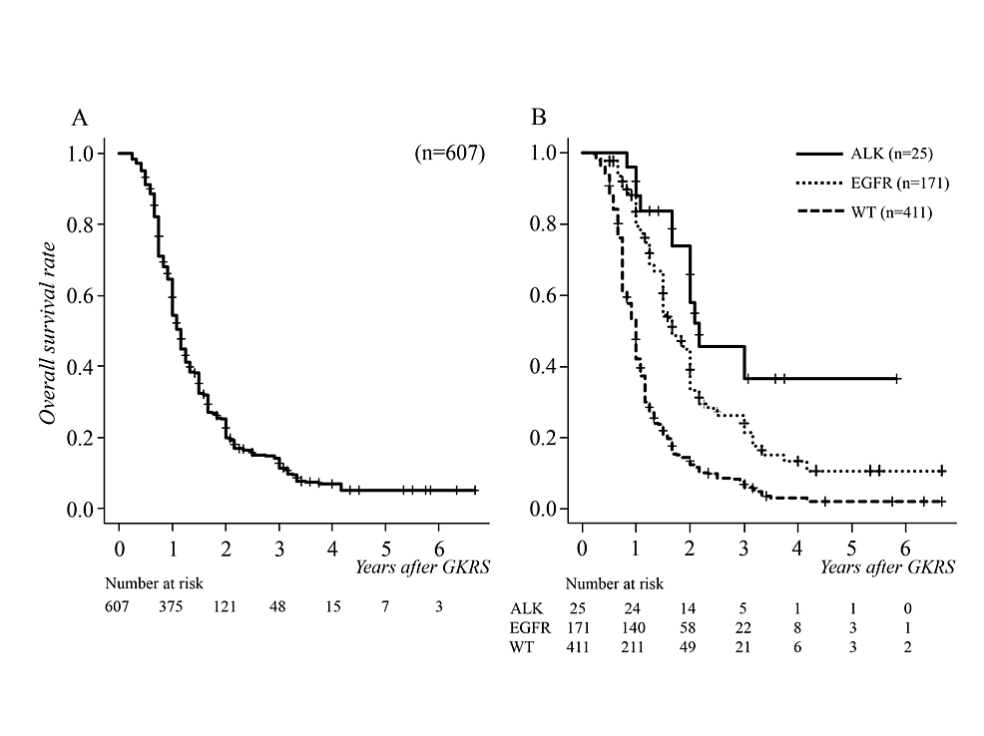
Kaplan-Meier survival curves for the overall survival rate following GKRS (A) The median overall survival time was 14 months. (B) There were statistically significant differences between driver mutation-positive (median 26 months in ALK-positive type and 20 months in EGFR-positive type) and wild types (median 12 months) (p < 0.0001), and ALK-positive and EGFR-positive types (p = 0.04). ALK, anaplastic lymphoma kinase; EGFR, epidermal growth factor receptor; GKRS, gamma knife radiosurgery; WT, wild type

In this study, all EGFR- or ALK-positive patients were receiving TKI medication at the time of treatment with GKRS. The median overall survival time after initial GKRS was 14 months (range 3-80, IQR 9-20, 95% CI 13-14). The overall survival rates at 6, 12, and 24 months after the initial GKRS were 91.1%, 54.4%, and 20.1%, respectively (Figure 1A).

Classified by driver gene mutations, median overall survival times after GKRS were 26 (95% CI 24-36) months in ALK-positive patients, 20 (95% CI 18-24) months in EGFR-positive patients, and 12 (95% CI 11-12) months in wild type patients. Driver gene mutation-positive patients survived significantly longer than wild type patients (p < 0.0001, HR 2.474, 95% CI 2.019-3.031), with a significant difference between ALK-positive and EGFR-positive patients (p = 0.04, HR 1.864, 95% CI 1.028-3.379) (Figure 1B).

Post-GKRS median overall survival times using the Lung-molGPA system were 26 months (95% CI 24-36) for patients with 3.5-4.0 points (56 patients), 18 months (95% CI 15-19) with 2.5-3.0 points (193 patients), 12 months (95% CI 12-13) with 1.5-2.0 points (263 patients), and 9 months (95% CI 8-9) with 0.0-1.0 points (95 patients). These four prognostic groups had significantly different median survival times (p < 0.0001, HR 1.804, 95% CI 1.616-2.015, log-rank test pooled overall strata). Post-GKRS median overall survival times using the M-RPA system were 20 months (95% CI 18­-23) for patients in Class 1 + 2a (242 patients), 12 months (95% CI 12-14) in Class 2b (128 patients), and 10 months (95% CI 9-11) in Class 2c + 3 (237 patients) (p < 0.0001, HR 1.761, 95% CI 1.588-1.953, log-rank test pooled overall strata). Multivariate analysis showed that older age, lower KPS score, multiple (five or more) intracranial metastases, uncontrolled primary cancer, uncontrolled extracranial metastases, no administration of immune checkpoint inhibitors, and negative driver gene mutation (wild type) cases were significantly correlated with unfavorable overall survival outcomes (Table 2).

**Table 2:** Prognostic variables affecting overall survival after GKRS CI, confidence interval; GKRS, gamma knife radiosurgery; HR, hazard ratio; KPS, Karnofsky Performance Status. *Log-rank test; **Cox proportional hazard model. †Significant difference at p < 0.05.

Variable (Tested for unfavorable outcome)	Univariate analysis*		Multivariate analysis**	
	p-value	HR (95% CI)	p-value	HR (95% CI)
Sex (male)	0.123	1.151 (0.9733-1.362)		
Age (≥70 years)	0.001†	1.336 (1.118-1.597)	0.003†	1.288 (1.072-1.548)
KPS score (<70%)	<0.0001†	1.752 (1.333-2.302)	0.0019†	1.581 (1.188-2.102)
No. of tumors at GKRS (≥5)	<0.0001†	1.366 (1.137-1.642)	0.001†	1.358 (1.123-1.643)
Primary cancer controlled (not)	<0.0001†	3.619 (2.993-4.376)	<0.0001†	2.724 (2.069-3.588)
Extra-cranial metastases (uncontrolled)	<0.0001†	2.267 (1.882-2.731)	<0.0001†	1.265 (1.196-1.665)
Immune checkpoint inhibitor (no)	0.04	1.416 (1.017-1.972)	0.001†	1.279 (1.051-2.080)
Driver gene mutation (wild)	<0.0001†	2.474 (2.019-3.031)	<0.0001†	2.553 (2.067-3.153)

**Figure 2:**
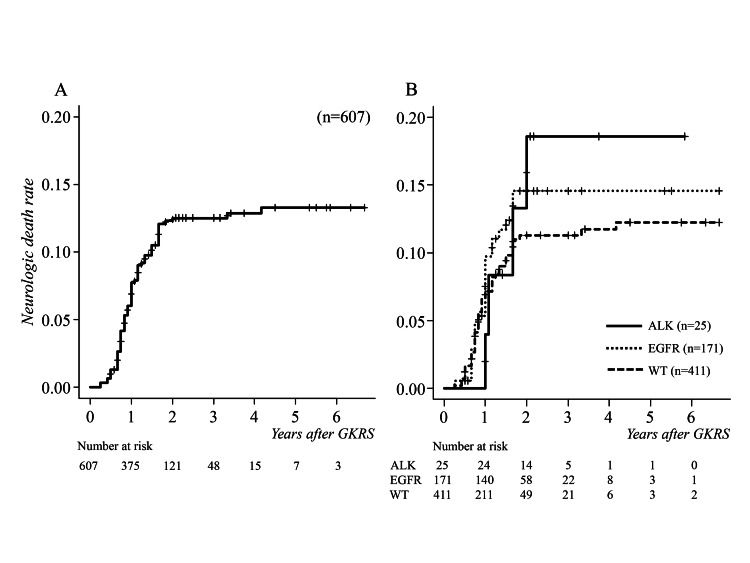
Cumulative survival curves for neurological death following GKRS (A) Median neurological survival time was 12 months. (B) There were no significant differences between driver gene mutation-positive and wild types (p = 0.33), and ALK-positive and EGFR-positive types (p = 0.83). ALK, anaplastic lymphoma kinase; EGFR, epidermal growth factor receptor; GKRS, gamma knife radiosurgery; WT, wild type

A total of 498 patients died during the follow-up period. The causes of death were systemic primary disease failure in 425 patients (85.3%) at a median of 12 months (range 3-50, IQR 9-18, 95% CI 12-13), and neurological disease failure in 73 patients (14.7%) at a median of 12 months (range 3-50, IQR 9-16, 95% CI 10-12). The latter included 71 patients with cerebral dissemination or carcinomatous meningitis and 2 with tumor recurrence. The cumulative neurological death rates were 1.2%, 7.9%, and 12.5% at 6, 12, and 24 months after initial GKRS, respectively (Figure 2A).

There were no significant differences between the presence or absence of driver gene mutations (p = 0.33, HR 1.261, 95% CI 0.788­­-2.017), and between ALK-positive and EGFR-positive patients (p = 0.83, HR 1.118, 95% CI 0.408-3.064) (Figure 2B).

**Figure 3:**
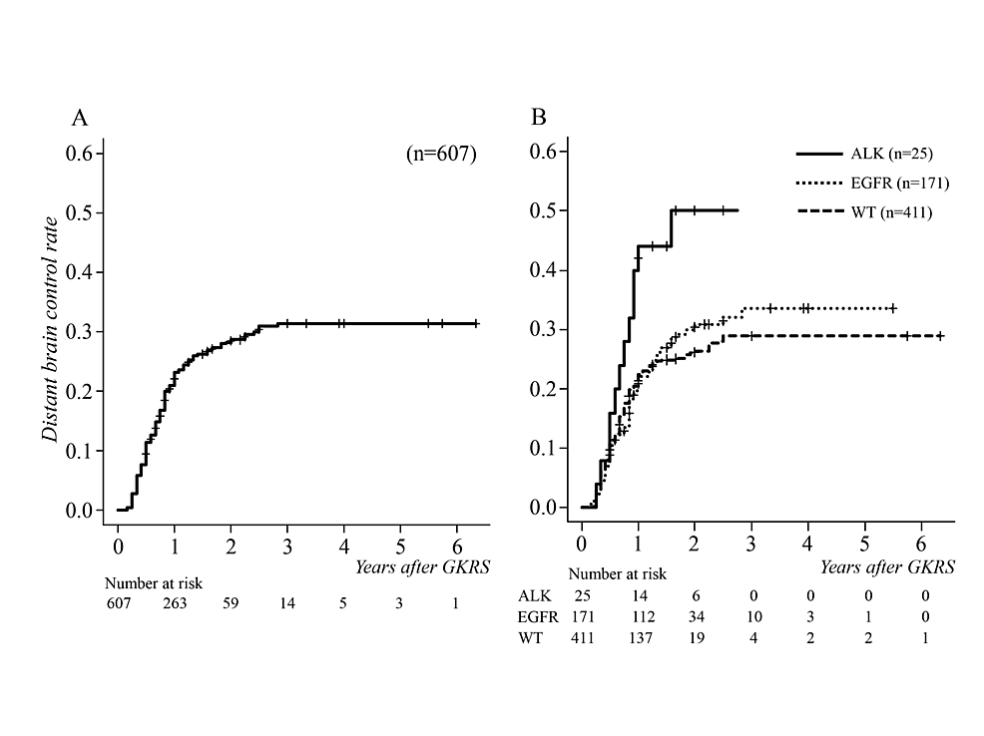
Cumulative survival curves for distant tumor control following GKRS (A) Median new brain metastases occurred at 15 months. (B) There was no significant difference between driver gene mutation-positive and wild types (p = 0.2), and a statistically significant difference between ALK-positive and EGFR-positive types (p = 0.047). ALK, anaplastic lymphoma kinase; EGFR, epidermal growth factor receptor; GKRS, gamma knife radiosurgery; WT, wild type

During the follow-up period after initial GKRS, new brain metastases appeared outside the GKRS-irradiated area at a median of 15 months (range 3-28 months, IQR 5-12 months, 95% CI 14-18) in 174 patients. Cumulative distant brain control failure rates were 11.4%, 23.3%, and 28.7% at 6, 12, and 24 months after initial GKRS, respectively (Figure 3A).

Additional treatments for new distant brain metastases were performed using salvage GKRS in 82 patients (median 6, range 2-6 times), and WBRT in 42. No significant difference was found between the presence or absence of driver gene mutations (p = 0.2, HR 1.217, 95% CI 0.899-1.647), but the incidence was significantly higher in ALK-positive patients than EGFR-positive patients (p = 0.047, HR 1.873, 95% CI 1.007-3.484) (Figure 3B).

Local tumor control of GKRS

A total of 2959 lesions (ALK-positive 191, EGFR-positive 938, wild type 1830) were treated with GKRS during the study period. Radiosurgical parameters at the time of GKRS are presented in Table 3.

**Table 3:** Radiosurgical parameters of GKRS-treated brain metastases from non-small cell lung cancer ALK, anaplastic lymphoma kinase; EGFR, epidermal growth factor receptor; GKRS, gamma knife radiosurgery; IQR, interquartile range. *Fisher’s exact test.

Characteristics	Value		
	Driver gene mutation positive	Wild type	p-value*
No. of tumors	1129 (ALK 191/EGFR 938)	1830	
Tumor volume			
Median (range, IQR), cm^3^	1.06 (0.23-9.01, 0.27-3.85)	1.85 (0.16-9.89, 0.33-4.29)	<0.0001
Prescription dose			
Median (range, IQR), Gy	20 (16-25, 18-22)	20 (16-25, 20-22)	0.082
Max dose			
Median (range, IQR), Gy	23.6 (16.9-53.5, 21.1-31.1)	25.1 (16.9-53.7, 22-36.1)	0.073
Tumor location, infra-/supratentorial, n	166/963	290/1540	0.432
Follow-up period			
Median (range, IQR), years	15 (3-80, 10-22)	9 (3-76, 6-12)	<0.0001

**Figure 4:**
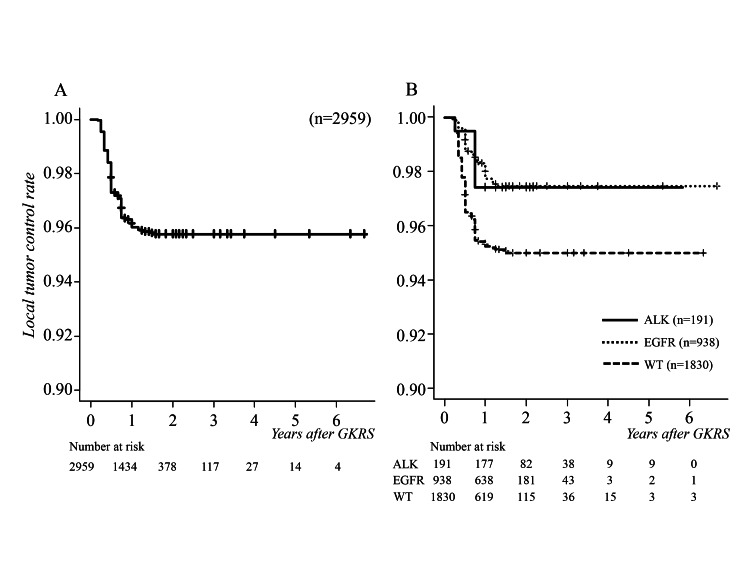
Cumulative survival curves for local tumor control following GKRS (A) Median tumor progression occurred at 12 months. (B) There was a statistically significant difference between driver gene mutation-positive and wild types (p = 0.001), and no difference between ALK-positive and EGFR-positive types (p = 0.95). ALK, anaplastic lymphoma kinase; EGFR, epidermal growth factor receptor; GKRS, gamma knife radiosurgery; WT, wild type

Follow-up neuroimaging showed complete response in 1101 tumors (37.2%), partial response and stable disease in 1738 (58.7%), and progressive disease in 120 (4.1%). The median time to tumor progression was 12 months (range 3-18, IQR 7-16, 95% CI 12-14). The cumulative local tumor control rates at 6, 12, and 24 months after GKRS were 97.3%, 96.1%, and 95.9%, respectively (Figure 4A).

Only five wild type patients required craniotomy due to uncontrolled tumor after GKRS. Tumors with positive driver gene mutations were significantly better controlled with GKRS compared to wild type tumors (p = 0.001, HR 2.051, 95% CI 1.346-3.125), with no significant difference between ALK-positive and EGFR-positive type tumors (p = 0.95, HR 0.973, 95% CI 0.370-2.555) (Figure 4B).

Radiation-related adverse events after GKRS

**Figure 5:**
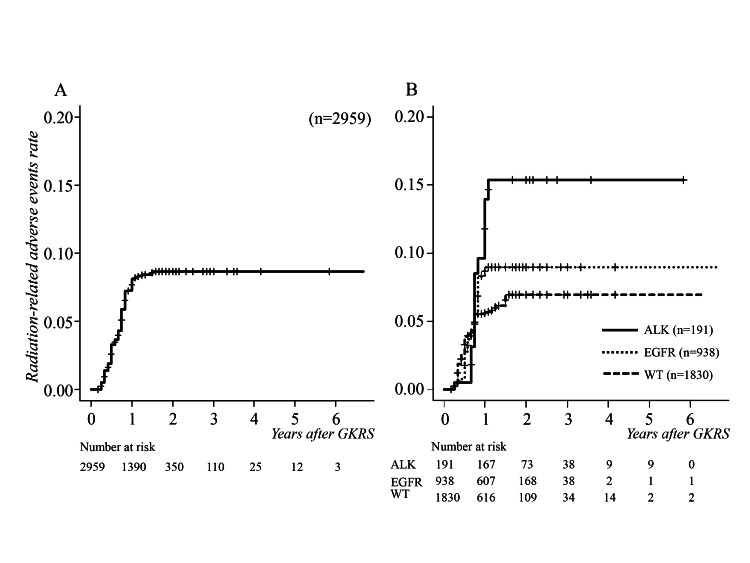
Cumulative survival curves for radiation-related adverse events following GKRS (A) Median adverse events related to GKRS occurred at 12 months. (B) There were statistically significant differences between driver gene mutation-positive and wild types (p = 0.021), and ALK-positive and EGFR-positive types (p = 0.02). ALK, anaplastic lymphoma kinase; EGFR, epidermal growth factor receptor; GKRS, gamma knife radiosurgery; WT, wild type

Adverse events related to GKRS occurred in 193 tumors (6.5%) at a median of 12 months (range 3-15, IQR 10-12, 95% CI 12-13) after GKRS. The cumulative GKRS-related complication rates were 3.3%, 8.1%, and 8.7% at 6, 12, and 24 months after initial GKRS, respectively (Figure 5A).

CTCAE late radiation morbidity grade was 2 in 203 tumors, and 3 in 82 tumors with motor weakness. The incidence was significantly higher in driver gene mutation-positive cases (p = 0.021, HR 1.378, 95% CI 1.049-1.809), and radiation damage occurred more frequently in ALK-positive than EGFR-positive tumors (p = 0.02, HR 1.650, 95% CI 1.083-2.512) (Figure 5B).

## Discussion

The present study demonstrated the therapeutic efficacy and safety of GKRS for brain metastasis from NSCLC with and without driver gene mutation based on our treatment experience. The main objective of this study was to compare the therapeutic effects of GKRS on ALK-positive patients with EGFR-positive and wild type patients, and to compare and verify the overall survival rate, incidence of neurological death, and development of new intracranial lesions after GKRS with and without driver gene mutations, as well as local tumor control and treatment-related complications caused by GKRS.

The overall long-term survival after GKRS tended to occur more in driver gene mutation-positive patients compared with wild type patients, similar to previous reports [10-12]. In particular, ALK-positive patients had significantly longer survival than EGFR-positive patients, associated with a trend toward a higher frequency of new intracranial brain metastases. The frequency of neurological death due to meningeal carcinomatosis was relatively higher in long-term survival in ALK-positive patients than in EGFR-positive and wild type patients, although the difference was not significant. Our study found the significant prognostic factors associated with longer survival were age, KPS score, number of brain metastases, control of the primary tumor, extracranial metastases, immune checkpoint inhibitors, and driver gene mutations.

Most of these factors are included in the parameters of Lung-molGPA, as the DS-GPA that considers driver gene mutations in cases of brain metastases from NSCLC, suggesting that our findings provided appropriate validation [15]. However, several issues were raised in this verification. The first is that ALK-positive patients were less common than EGFR-positive and wild type cases, limiting simple comparisons in terms of survival and the appearance of new intracranial brain metastases. Therefore, experience with more ALK-positive cases in the future is required for verification. The second is the effect of the chemotherapeutic drugs on the primary tumor. Unfortunately, this study did not adequately compare the use of TKIs during and after GKRS and did not fully consider the effect of TKI treatment on survival. Consequently, detailed verification of the use of TKIs and chemotherapeutic drugs will be necessary after accumulating more cases [1,3]. Other factors requiring evaluation include the effect of TKI use after GKRS, discontinuation of TKIs due to drug resistance, concomitant use of other drugs, and drug switching on the appearance of new intracranial lesions after GKRS. In particular, the evaluation of differences in the effects of each generation of TKIs used over time on brain metastases in patients with long-term survival is necessary [1,3]. In addition, this study found that the use of immune checkpoint inhibitors, which is expected to increase in the future, was a prognostic factor significantly related to survival, and should be considered in the future [1].

Patients with the driver gene-mutant cases showed significantly acceptable outcomes for local tumor control after GKRS compared to the wild type cases in this study. Radiation-related adverse events also tended to occur more frequently in patients with positive driver gene mutations. In particular, ALK-positive patients had the highest incidence of treatment-related complications, although the number of cases was small. Previously, the incidence of radiation injury was reported as higher in patients with positive driver gene mutations, especially in patients using TKIs, due to the effect of increased radiosensitivity [17,18]. The present study demonstrated that radiation-related adverse events occurred at a higher rate in driver gene mutation-positive patients, especially in about 15% ALK-positive patients almost one year after GKRS.

Local control of the tumor is very likely to influence the improvement of neurological symptoms observed at the time of GKRS and the prevention of new neurological symptoms in the foreseeable future. The maintenance and improvement of performance status will influence the continuation of chemotherapy. Therefore, it is important to continue to perform regular intracranial imaging whenever possible and to aggressively perform early GKRS if new or recurrent brain metastatic lesions are detected, especially in patients with long-term survival. However, we recommend to consider the possibility of radiation damage and reduce the radiation dose in driver gene mutation-positive patients, especially ALK-positive patients. In addition, the efficacy of TKIs for local control of brain metastases has been extensively discussed [1,3,6-8]. Therefore, the efficacy of only GKRS in treating brain metastases may be difficult to discuss if TKIs are used during and after GKRS. However, considering the problem of drug resistance to TKIs in the future, we consider that active performance of GKRS to obtain local tumor control in patients with long-term survival is effective, while considering the treatment-related complications.

This study indicated that the therapeutic effect of GKRS in patients with NSCLC harboring driver gene mutations should be investigated in terms of survival, neurological death, appearance of new intracranial lesions, local tumor control, and development of radiation injury. This study has many issues, but we believe that GKRS is effective for the control of brain metastases because many patients with positive driver gene mutations, especially ALK-positive patients, are expected to survive for a long time after GKRS and have a good prognosis in terms of tumor control.

## Conclusions

Many patients with brain metastases from ALK rearrangement-positive and EGFR mutation-positive NSCLC achieve relatively long-term survival after GKRS, and ALK-positive patients tend to have significantly longer survival. Consequently, ALK-positive patients tend to have a higher incidence of new intracranial lesions due to the long-term survival, associated with a relatively higher incidence of neurological death due to meningeal carcinomatosis. GKRS shows significantly satisfactory local tumor control in driver gene mutation-positive patients. On the other hand, driver gene mutation-positive patients, especially ALK-positive patients, tend to have a higher incidence of radiation injury, suggesting the need for periodic imaging follow-up after GKRS.
